# Analysis of Immune Microenvironment by Multiplex Immunohistochemistry Staining in Different Oral Diseases and Oral Squamous Cell Carcinoma

**DOI:** 10.3389/fonc.2020.555757

**Published:** 2020-12-08

**Authors:** Bin Qiao, Junwen Huang, Zi Mei, Alfred King-yin Lam, Junfang Zhao, Le Ying

**Affiliations:** ^1^ Department of Oral and Maxillofacial Surgery, The First Affiliated Hospital of Zhengzhou University, Zhengzhou, China; ^2^ Cancer Molecular Pathology and Griffith Medical School, Griffith University, Gold Coast, QLD, Australia; ^3^ Centre for Innate Immunity and Infectious Diseases, Hudson Institute of Medical Research, Clayton, VIC, Australia

**Keywords:** oral squamous cell carcinoma, multiplex immunochemistry, PD-L1, oral disease, CD8

## Abstract

**Purpose:**

The aim is to investigate the impacts of using multiplex immunochemistry (mIHC) staining to analyses the co-expression of programmed death ligand-1 (PD-L1) and tumor infiltrating lymphocytes (TILs) [CD8^+^ T cells and Forkhead Box Protein 3 (FOXP3)^+^ regulatory T cells (Tregs)] in different oral diseases, and oral squamous cell carcinoma (OSCC).

**Methods:**

Formalin fixed paraffin-embedded tissue sections from different oral diseases were stained with PD-L1 and TILs (CD8^+^ T cells and FOXP3^+^ Tregs) by mIHC staining simultaneously. The whole slide was scanned digitally to observe the cell phenotypes stained in the microenvironment. The contents of each slice were read using a computer-aided method to analyze and the cell densities were calculated using statistical software.

**Results:**

We were able to characterize the tumor microenvironment (TME) of different oral diseases including oral leukoplakia (OLK), inflammatory gingiva (IG), oral lichen planus (OLP), and squamous cell carcinoma (SCC), with accurate visualization of various immune cells harboring complex immune phenotypes by mIHC staining. The results showed that PD-L1 was up-regulated in SCC tissues at different pathological stages, while CD8 and FOXP3 had no significant changes. The ratio of PD-L1/CD8 was also significantly up-regulated in SCC tissues compared with that of other oral diseases. In advanced stages of OSCC, the FOXP3/CD8 ratio increased, and immunosuppressive TME was more pronounced. In addition, we also found different immune phenotypes: the inflamed phenotype, immune-excluded phenotypes, and immune-desert phenotypes. By locating tumor epithelial cells, we found that PD-L1 expression is in both tumor cells and stromal cells.

**Conclusions:**

mIHC is useful for the visualization and evaluation of tumor microenvironment in immuno-oncology research. It allows single-cell imaging *in situ* and could effectively and quickly determine the immune phenotype of different oral diseases.

## Introduction

Oral cancer is one of the most common malignant tumors of the upper gastrointestinal system in the world, among which SCC is the most common pathological type, accounting for about 90% of all oral squamous cell carcinomas (OSCCs) (1). Despite advances in chemotherapy, targeted radiotherapy, and surgery, patients with terminal cancer still have poor prognosis (5-year survival rate: 50%) (2). Surgical treatment combined with radiotherapy and chemotherapy is still the main treatment. Although the progress of OSCC treatment has improved the efficacy of some patients in recent years, the prognosis is still not satisfactory (3). Therefore, in-depth understanding and diagnosis of the pathological types of OSCC can help us further stratify patients into certain subtypes, which may favor these patients with more effective treatment methods.

TILs were reported for the first time in 1986 by Rosenberg (4), to play a vital role in cancer immunotherapy. The phenotypes of TILs are heterogeneous. Most of TILs are CD3^+^ T cells, which are predominately CD8^+^ cytotoxic T cells. Moreover, high numbers of CD8^+^ T cells are positively correlated with the patients’ low lymph node metastasis rates, tumor recurrence rates, and longer survival times in various human cancers, such as melanoma (5), ovarian carcinoma (6), and head/neck squamous cell carcinoma (HNSCC) (7). There is increasing evidence that different numbers and types of TILs are found in different sites, such as within or around tumor tissue. In fact, they are not randomly distributed, they’re located in specific areas (8). Therefore, the location of CD8^+^ T cells may have an impact on the prognosis of patients with OSCC.

Immune checkpoints (ICPs) are a series of inhibitory signaling pathways that regulate the immune system. Under normal physiological conditions, ICPs have great significance in maintaining immune tolerance, regulating immune response, and preventing self-tissue damage. However, in the tumor process, the activation and high expression of ICPs can inhibit the function of immune cells and mediate tumor immune escape (9). Programmed death receptor ligand 1 (PD-L1) is an important negative co-stimulatory molecule that mediates the immunosuppressive function of tumor microenvironment. The abnormal expression of PD-L1 in tumor tissues is closely related to patients’ clinical pathological parameters and prognosis. PD-L1 and CD8^+^ TILs, as important factors of anti-tumor immune response, often determine the efficacy of anti-programmed death receptor 1 (PD-1)/PD-L1 antibodies in various solid tumors. Therefore, understanding the expression and proportional relationship between PD-L1 and CD8^+^ TILs in oral diseases and SCC is helpful for research and development of more effective immune checkpoint blocking therapy. Defining the expression density of PD-L1 and CD8^+^ TILs is helpful to determine the prognosis of SCC patients, and it can also accurately select the subgroup that would benefit the most from anti-PD-1/PD-L1 antibody therapy (10). Immunotherapy will re-energize and reinforce the existing anti-tumor immune response in the human body. Conversely, patients with a lack of PD-L1 in TILs were observed to have unobstructed tumor progression, suggesting that PD-L1 blockade neither promotes CD8^+^ T cell immunity nor prevents effective T cells from infiltrating tumors (11).

CD8^+^ T cells are regulated by Tregs, which secrete several suppressive cytokines, such as transforming growth factor beta (TGF-β), to regulate activation and proliferation of CD8^+^ T cells (12). FOXP3 is a specific marker of CD4^+^ Tregs and have been linked to OSCC progression. Immunoregulatory therapy reduces immunosuppressive signals in OSCC patients can improve the prognosis of patients.

Overall, studying the tissue location and infiltration of CD8^+^ T cells, PD-L1^+^ cells, and FOXP3^+^ Tregs in patients with OSCC has important clinical significance for evaluating the treatment plans and predicting the prognosis of patients. Traditional immunohistochemical stained section could not detect interaction of different subtypes of tumor infiltrating cells in a single section. mIHC staining solves this problem by immunostaining various markers on the same slide and calibrating digital images using computer software to evaluate multiple parameters of the cell lineage, histological localization of individual tumor and immune subpopulations. The technology can maximize the information from limited tissues, and can also display the tissue anatomical relationships of different cytokines in the peritumoral and intratumoral chambers (13).

In this study, we aim to investigate the advantages of four-color mIHC method to detect the expression and localization of CD8, PD-L1, and FOXP3 expression cells in different oral diseases and oral squamous cell carcinoma.

## Material and Methods

### Human Samples

Human oral tissues were obtained from the Department of Oral and Maxillofacial Surgery of the First Affiliated Hospital of Zhengzhou University (Zhengzhou, China) between 2017 and 2019. These samples were fixed in formalin and embedded in paraffin. The use of human specimens was approved by the Oral and Maxillofacial Surgery of the First Affiliated Hospital of Zhengzhou University, and the patient’s informed consent was obtained. Forty oral tissue samples were used in this study, including 13 OLK tissues, 3 IG tissues, 7 OLP tissues, and 17 SCC tissues. All the tissues have been sectioned and stained with hematoxylin and eosin (H&E) staining to double check the pathology type of each sample.

### Immunohistochemistry Staining

Optimization of IHC staining is based on the protocol we used in our previous studies (14, 15) All the human oral tissues were sectioned into 4 um-thick slides. The slides were dewaxed in three times of xylene, three times of 100% ethanol, and followed by a serial of ethanol (70, 50, and 25%). Freshly made citrate buffer (pH = 6.0) was used in the antigen recovery step. The slides were microwaved with the citrate buffer for 15 min. Sample endogenous peroxidase is inactivated after 10 min incubation at 3% H_2_O_2_ at room temperature. After blocking the tissues for 1 h using blocking serum, the primary antibodies [CD8, FOXP3, and PD-L1(78701T), Cell Signaling Technology, Danvers, MA, USA] was applied and incubated for 1 h at room temperature. Then, the slides were washed three times with this buffered saline (TBST) (3 min each). After that, all the slides then incubated according secondary antibody, horseradish peroxidase (HRP) and ABC reagent (Vectastain ABC kit, Vector Laboratory Ltd., Burlingame, CA, USA). Then, DAB was dyed with 3,3’-diaminobenzidine (DAB), hematoxylin was re-dyed and fixed with dibutyl phthalate polyphenylene (DPX) (Sigma, St. Louis, MO, USA). All staining included an isotype control. The concentration of primary antibodies was optimized using oral tissues and we applied 1:500 dilution for CD8, 1:450 dilution for FOXP3, and 1:350 dilution for PD-L1 for oral tissue staining in this study.

### Multiplex Immunohistochemistry Staining

For the mIHC staining in this study, the same primary antibodies [CD8, FOXP3 and PD-L1 (78701T), Cell signaling, USA], as well as an Opal four-color fluorescent IHC kit (PerkinElmer, Waltham, MA, USA) were used as our previous studies (14, 15). We used Opal 520 channel for PD-L1 [fluorescein isothiocyanate (FITC), a green fluorescence stain], Opal 570 channel for FOXP3 [cyanine 3 (Cy3), an orange fluorescence stain], Opal 670 channel for CD8 [cyanine 5 (Cy5), a red fluorescence stain], and DAPI (4′,6-diamidino-2-phenylindole, a blue fluorescence stain). All the 40 slides were observed and imaged by Olympus FV1200 confocal system (Tokyo, Japan) first and for some of the slides, we used a Nikon C1 confocal system (Tokyo, Japan) to do a whole tissue scanning. All the images were analyzed by ImageJ software (NIH, Bethesda, MD, USA). The number of CD8^+^ and FOXP3^+^ cells was calculated by means of particle analysis tool and the intensity of PD-L1 in each region was determined by ImageJ software. Since there was much deviation, we did a log10 calculation based on the value we got from ImageJ.

### Statistical Analysis

Statistical analysis used Kruskal–Wallis test to perform multiple comparisons to GraphPad Prims (Version 7.0, San Diego, CA, USA), and differences were considered significant when p < 0.05.

## Results

### Contrast Staining (IHC/mIHC) of Different Markers in Oral Cancer (Tongue Cancer Tissues)

**Figure 1:**
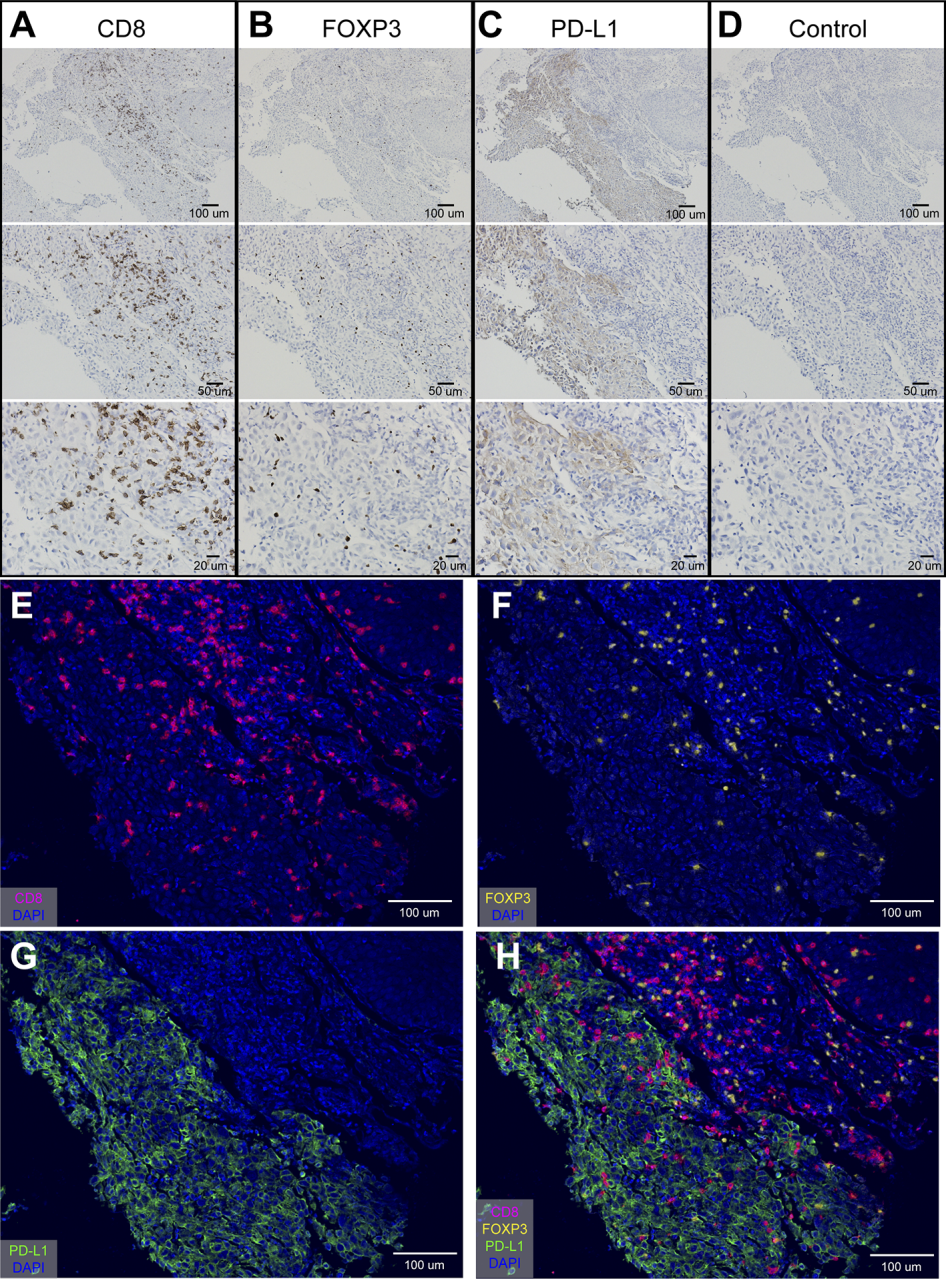
Optimization of IHC staining and four-color mIHC staining of paraffin-embedded oral cancer (tongue cancer) tissue. **(A)** IHC staining of CD8; **(B)** IHC staining of FOXP3; **(C)** IHC staining of PD-L1; **(D)** IHC staining of control IgG in oral cancer tissue; **(E–H)** Four-color mIHC staining of CD8 **(E)**, FOXP3 **(F)**, PD-L1 **(G)**, and merge image **(H)** in a serial oral cancer tissue. DAPI was used to visualize nuclei (blue color).

mIHC staining accurately described the microenvironment in multiple OSCC regions. Firstly, we use the tongue squamous cell carcinoma tissue at the same place, and use traditional immunohistochemical methods to optimize the experimental conditions of oral cancer tissue. After optimization, we can clearly see specific expression of CD8^+^ T cells, PD-L1^+^, cells and FOXP3^+^ T cells in the tissue. We noted that CD8 (**Figures 1A, E**) and FOXP3 (**Figures 1B, F**) expressed in TILs, while PD-L1 expressed in both tumors and TILs (**Figures 1C, G**). **Figure 1D** showed IHC staining of control IgG. We can clearly see that CD8 stained on the surface of lymphocytes (**Figures 1A, E**) and FOXP3 in the nuclear of the lymphocytes (**Figures 1B, F**).

Besides, studies have shown that the expression of PD-L1 in infiltrating stromal cells is more common and even more predictive of the response than the expression of PD-L1 in individual tumor cells (16). Tumor stromal cells mainly consist of some infiltrating lymphocytes and other non-tumor cells. In our study, we found that PD-L1 were highly expressed in the membrane and/or cytoplasm of tumor and stromal cells (**Figures 1C, G**). The differences in the immune background of multiple regions in oral tongue cancer are shown in **Figure 1H**. These results suggest that in oral cancer tissues, the high expression of PD-L1 is likely to be a marker of disease prognosis.

### Quantification of PD-L1 Is Meaningful in the Diagnosis of Different Oral Diseases Samples

OLK is the most common oral precancerous lesion, with 3 to 5% of OLK converted to OSCC (17). IG is a chronic infectious inflammatory disease caused by a large number of inflammatory invasions. OLP is a common oral mucosal streak disease, which is related to autoimmune or systemic diseases and has a tendency to become cancerous.

**Figure 2:**
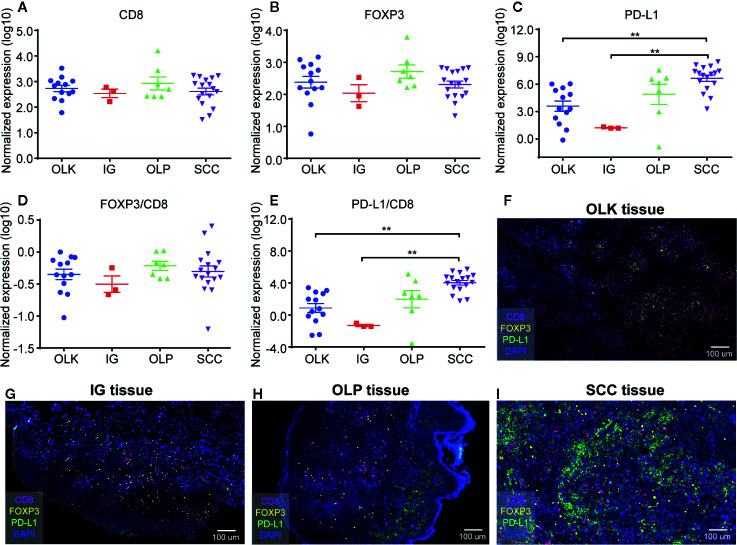
Quantification of CD8^+^ T cells, FOXP3^+^ Tregs, and PD-L1^+^ cells in 40 different oral diseases samples. **(A)** Normalized expression (log10) of the number of CD8^+^ T cells per mm^2^; **(B)** Normalized expression (log10) of the number of FOXP3^+^ Tregs per mm^2^; **(C)** Normalized expression (log10) of the intensity of PD-L1^+^ cells per mm^2^; **(D, E)** Ratio of normalized expression of FOXP3/CD8 **(D)** and PD-L1/CD8 **(E)**; **(F)** Representative image of lesions-oral leukoplakia (OLK) tissue; **(G)** Representative image of inflammatory gingival (IG) tissue; **(H)** Representative image of OLP tissue; **(I)** Representative image of SSC tissue. Data were analyzed using the Kruskal-Wallis test (multiple comparisons). **P < 0.01.

Statistical analysis of the expression of CD8^+^ T cells (**Figure 2A**), FOXP3^+^ cells (**Figure 2B**), and PD-L1^+^ cells (**Figure 2C**) in different oral lesions. There was no significant difference on the expression of CD8 and FOXP3 among different tissue types (**Figures 2A, B**). On the other hand, the expression of PD-L1 was significantly increased in SCC tissues, compared to OLK and IG tissues (**Figure 2C**). These results indicated that the quantification of PD-L1 is meaningful in the diagnosis of squamous cell carcinoma, inflammatory diseases, and precancerous lesions.

The density of TILs, PD-L1, and CD8 cells in the tumor determined the prediction of the tumor microenvironment response to immunocheckpoint inhibitors (18, 19). The proportion of FOXP3^+^ Tregs and tumor infiltrating CD8^+^ T cells had positive relationship with patient prognosis and immune escape. The higher ratio, the worse prognosis and the stronger immune escape ability (20), while low ratio of FOXP3^+^/CD8^+^ was identified as independent factors to predict the response to PD-1 blockade therapy (21). However, in this study, we found that the ratio of FOXP3^+^ Tregs/CD8^+^ T cells has no significant differences among these oral diseases (**Figure 2D**).

Since PD-L1 expression is not a predictor of PD-1 blocking response, assessing the ratio of PD-L1 to TILs is a key factor in precisely predicting immune checkpoint inhibitor response in patients with high/low PD-L1 expression, which can compensate for PD -L1 expression limitations (21). In this research, statistical comparison showed that PD-L1/CD8 ratio difference between squamous cell carcinoma and other oral diseases (OLK, IG) was statistically significant (**Figure 2E**). Therefore, we can speculate that the PD-L1/CD8 ratio is higher, OSCC may have an effective clinical response to anti-PD-1/anti-PD-L1 treatment.

Next, we further demonstrated this argument by analyzing the sections of different oral diseases (**Figures 2F–I**). There were more CD8^+^ T cells and PD-L1^+^ cells expression in pre-malignant oral lesions (OLK or OLP) than that of inflammatory gingivitis. In addition, the highest PD-L1 expression was noted in OSCC among these oral diseases.

### Quantification of Different Markers Has Significance in the Diagnosis of Clinical Stage of Oral Cancer

**Figure 3:**
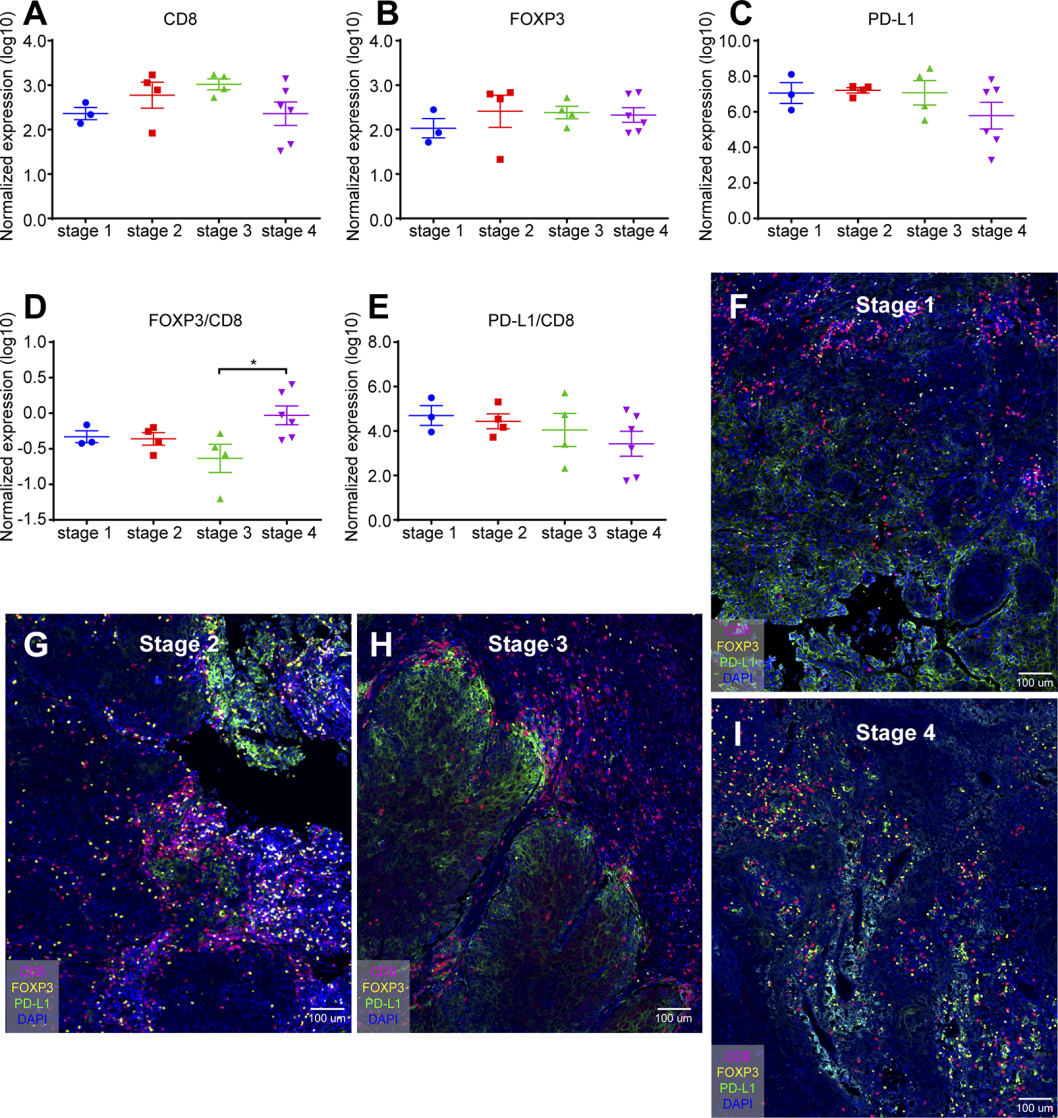
Quantification of CD8^+^ T cells, FOXP3^+^ Tregs, and PD-L1^+^ cells in different stage of oral cancer samples. **(A)** Normalized expression (log10) of the number of CD8^+^ T cells per mm^2^; **(B)** Normalized expression (log10) of the number of FOXP3^+^ Tregs per mm^2^; **(C)** Normalized expression (log10) of the intensity of PD-L1^+^ cells per mm^2^; **(D, E)** Ratio of normalized expression of FOXP3/CD8 **(D)** and PD-L1/CD8 **(E)**; **(F)** Representative image of SSC sample from stage 1; **(G)** Representative image of SSC sample from stage 2; **(H)** Representative image of SSC sample from stage 3; **(I)** Representative image of SSC sample from stage 4. Data were analyzed using the Kruskal-Wallis test (multiple comparisons). *p < 0.05.

Tumor stage is not only a reliable indicator for accurately predicting the biological behavior and prognosis of malignant tumors, but also a prerequisite for clinicians to choose a suitable treatment plan and improve the treatment effect (22). Our study found that the normalized expression of CD8 (**Figure 3A**), FOXP3 (**Figure 3B**), and PD-L1 (**Figure 3C**) at different OSCC stages was not statistically significant (Log10). In addition, we examined the FOXP3/CD8 ratio (**Figure 3D**) and the PD-L1/CD8 ratio (**Figure 3E**). FOXP3/CD8 ratio was statistically significant only in stages 3 and 4, and no statistical difference was found in other stages (**Figure 3D**). There was no difference in PD-L1/CD8 at any stage (**Figure 3E**).

In addition, mIHC staining were used to verify the expression patterns of these three key elements in different stages. **Figure 3F** was from the cheek carcinoma of stage 1, **Figure 3G** was from the maxillary squamous cell carcinoma of stage 2, **Figure 3H** was from the maxillary squamous cell carcinoma of stage3, and **Figure 3I** was from the tongue carcinoma of stage 4. Infiltration of CD8^+^ T cells was more in stages 2 and 3 (**Figures 3G, H**), and the expression of PD-L1 was more in stage 3 (**Figure 3H**), which was consistent with the statistics of **Figure 3C**. On the other hand, FOXP3 showed no significant difference in expression at different stages of OSCC. These results indicate that the quantification of different markers is of great significance for the diagnosis of clinical stage of oral cancer.

### Different Markers Expressed Differently in Different Immune-Phenotype Samples

**Figure 4:**
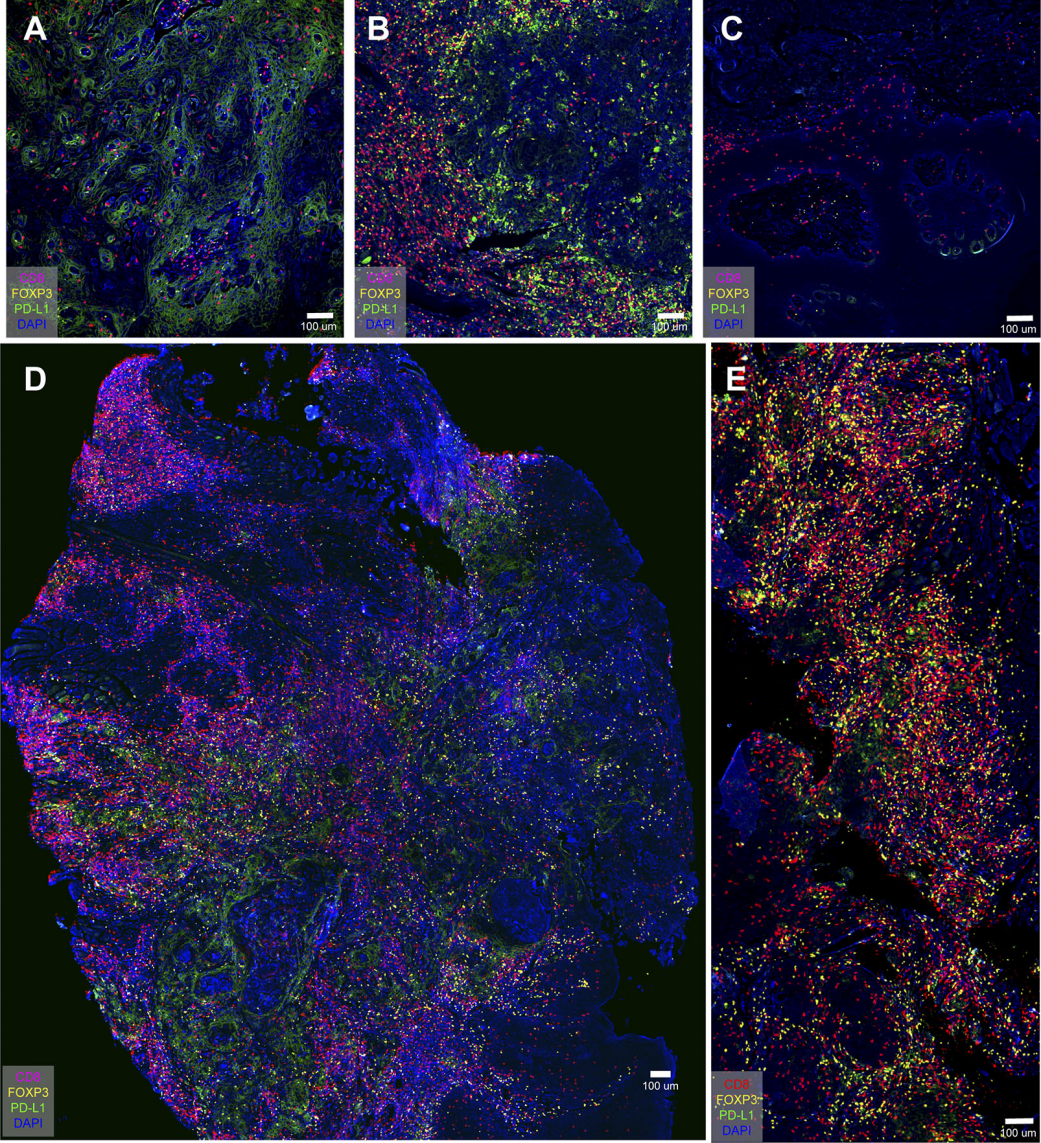
PD-L1 distribution in oral cancer samples and overview immune profiling of tongue cancer and cheek cancer tissue. **(A)** PD-L1 widely spreads across the oral tissue; **(B)** PD-L1 locally restricts to the certain area of the oral tissue; **(C)** PD-L1 barely expresses in the oral tissue; **(D)** The distribution of CD8^+^ T cells, FOXP3^+^ Tregs and PD-L1^+^ cells in tongue cancer tissue; **(E)** The distribution of CD8^+^ T cells, FOXP3^+^ Tregs and PD-L1^+^ cells in cheek cancer tissue.

Three cases (all stage 4) were selected to demonstrate the inherent immunological status—or “cancer-immune set point”—of an individual. **Figure 4A** was an “inflamed phenotype” TME from gingiva carcinoma, and the PD-L1 expression was widely and evenly noted across the squamous cell carcinoma. CD8^+^ T cells and FOXP3^+^ Tregs were present in the inflammatory cells infiltrating between the carcinoma cells. **Figure 4B** was an “immune-excluded phenotype” from tongue cancer and PD-L1 locally restricted to a certain area of the oral tissue. The stromal CD8^+^ cells and Treg^+^ cells did not penetrate between the tumor cells. **Figure 4C** was an “immune-desert phenotype” from a tongue carcinoma. PD-L1 barely expresses in the carcinoma. CD8^+^ cells and Tregs were not detected. These results demonstrated the difference in tumor microenvironment of carcinoma of same histological type in different patient. **Figure 4D, E** were two whole tissue scanning images from different patients, indicating the heterogeneous microenvironments in patients with the same type of cancer.

### Lymphocytes Free From Tumor Tissue and PD-L1 Infiltration

**Figure 5:**
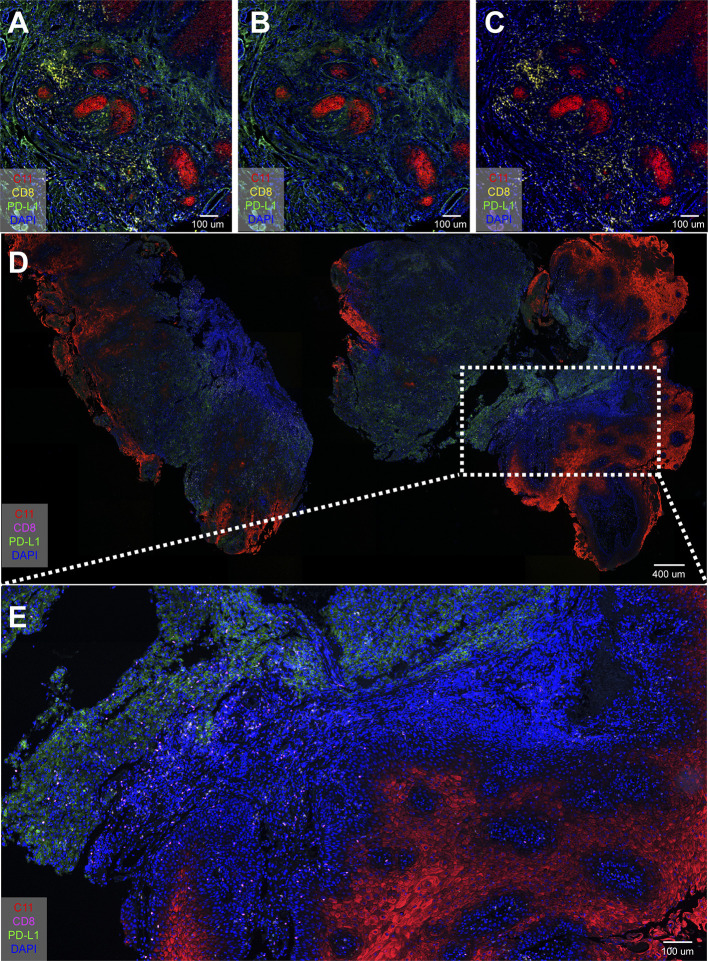
Spatial localization among CD8^+^ T cells, PD-L1^+^ cells, and epithelial cells. **(A–C)** Four-color mIHC staining of Pan-keratin (C11) cells, PD-L1^+^ cells, and epithelial cells in oral cancer tissue; **(D, E)** Overview expression of CD8^+^ T cells, PD-L1^+^ cells, and epithelial cells in tongue cancer tissue.

Pan-cytokeratin (pan-CK/C11) is the most common marker in epithelial cell differentiation (23). **Figure 5** uses C11 to clearly show the spatial relationship between CD8^+^ cells, PD-L1 and tumor epithelial cells. **Figure 5A** was a merge of **Figures 5B, C**. CD8^+^ T cells did not infiltrate between the tumor tissues (**Figure 5B**), while PD-L1 could be clearly seen in the tumor tissues (**Figure 5C**). **Figures 5D, E** were whole-tissue scanning images from tongue cancer tissue of stage 3 which overview expression of CD8^+^ T cells, PD-L1^+^ cells, and epithelial cells. These results confirmed that in advanced OSCC, CD8^+^ T cells were mostly detected outside the tumor tissues, but did not penetrate between the tumor cells, so the tumor could not be eliminated by the patient’s own immunity.

## Discussion

In this study, we used four-color mIHC technology to visually describe the immune environment of human oral diseases. Our data suggest that due to less infiltration of CD8^+^ T cells and more expression of FOXP3 and PD-L1, the interaction between different immune populations can be used as an important factor to stratify patients and achieve individualized precision medicine in the immunosuppressive tumor microenvironment of OSCC.

In addition, we use quantitative method to evaluate the expression of TILs. Although different tumor sampling strategies may produce inconsistent results in terms of the lymphocyte density of tumor infiltration, random sampling of tumor centers, or large nucleus biopsy (10 × 1 mm), may best represent the entire tumor (24). In **Figures 2** and **3**, we counted the numbers of these markers in different oral diseases (OLK, OLP, IG) and stages/mm^2^. PD-L1 expression is significantly higher in advanced OSCC than in other oral diseases and SCC stages.

The ratio of the FOXP3/CD8 and PD-L1/CD8 as well as the density of TILs, these are feasible ways to understand the mechanism of action of treatment and to predict the response to treatment. Based on our results, FOXP3^+^ tumor infiltrating lymphocytes in OSCC may have a similar inhibitory effect as other solid tumors by inhibiting the cytotoxic activity of CD8^+^ T cells and affecting the response to immunotherapy. The number of CD8^+^ cells is negatively correlated with the immune expression of PD-L1, suggesting that tumor infiltrating CD8^+^ cells may have other functions besides inhibition. *In vitro* coexistence of Tregs suppresses the nivolumab-induced release of interferon-γ from effector T cells (25). In general, Tregs may interfere with the immune stimulation of PD-L1 inhibitors in OSCC.

By analyzing the immunomarker PD-L1, we found that the absence of PD-L1 expression and the cancer cell mesenchymal phenotype were present in some highly invasive human OSCC tissue (**Figure 4C**). Interestingly, strong PD-L1 signaling was detected in stromal cells in highly invasive human OSCC tissue (stage 3, **Figure 5E**). Previous reports have provided evidence that PD-L1 is expressed not only by tumor cells but also by tumor-related macrophages, dendritic cells, and cancer associated fibroblasts (26). Despite the level of PD-L1 expression in tumor cells, tumor-infiltrating immune cells have recently been shown to be associated with clinical responses to anti-PD-1 therapy (27).

However, only a part of the tumor patients with PD-L1 expression showed a clinical response, while other patients (without PD-L1 staining) showed clinical benefit, indicating that there may be other factors in the tumor microenvironment (such as the expression of PD-L1 in stromal cell), which affected the benefits patients. The complex tumor microenvironment is difficult to be analyzed with CD8, PD-L1 and other single markers. Using multi-parameter analysis of immune checkpoints such as PD-L1, CD8, and FOXP3 to study the interactions between cell types can provide a more comprehensive immune phenotype in the tumor microenvironment and help predict prognosis and precise treatment.

In this study, PD-L1 is mostly present outside tumor cells area and this is not accordant with many previous reports that PD-L1 mainly expressed on tumor cells. According to the spatial positioning of these markers, the relative spatial distribution of PD-L1 in tumor microenvironment might be an important factor to stratify patients for personalized precision medicine.

These findings and our study revealed that the tumor microenvironment in the complex interaction, and emphasizes the role of different types of immune cells in the tumor microenvironment may be highly dependent on many factors is the key to tumor phenotype and function of it is worth noting that not only the number of TILs type and location, and the activity of specific infiltrating cells (such as cytokine release mode) can define correlation and prognostic value. Therefore, it is best to comprehensively evaluate the expression of PD-L1, FOXP3, and CD8^+^ T cells by mIHC for evaluating interactions of these cells.

## Conclusion

With further development of cell segmentation, cell intensity quantification, and high-throughput image analysis methods, mIHC staining can play a more important role in predicting response to immunotherapy. These results will help determine the prognostic response of PD-1/PD-L1 inhibitors to oral cancer patients. In addition, the study of interaction of different cells in different pathological stages may ultimately help physicians to choose a reasonable initial and sequential treatment plan for each patient.

## Data Availability Statement

The raw data supporting the conclusions of this article will be made available by the authors, without undue reservation.

## Ethics Statement

The studies involving human samples were reviewed and approved by Research Ethics Committee of the First Affiliated Hospital of Zhengzhou University

## Author Contributions

JZ and LY designed the study and conducted critical revision of the manuscript. BQ and JH conducted acquisition, analysis, and interpretation of the data. ZM wrote the manuscript and AL revised it. All authors contributed to the article and approved the submitted version.

## Funding

Health Commission of Henan Province, China (Grant NO. SB201902006), supported this research.

## Conflict of Interest

The authors declare that the research was conducted in the absence of any commercial or financial relationships that could be construed as a potential conflict of interest.
